# Male breast carcinoma: radiotherapy contributed to favorable local control in two cases and related literature review

**DOI:** 10.1186/s40001-015-0173-x

**Published:** 2015-11-26

**Authors:** Min Liu, Qiang Wang, Bin Liu, Ling Gao, Di Wu, Shuo Yang, Bailong Liu, Lihua Dong

**Affiliations:** Department of Radiation Oncology, The First Hospital, Jilin University, 71 Xinmin Street, 130021 Changchun, China; Department of Hand Surgery, The First Hospital, Jilin University, 71 Xinmin Street, 130021 Changchun, China; Department of Breast Surgery, The First Hospital, Jilin University, 71 Xinmin Street, 130021 Changchun, China

**Keywords:** Male breast carcinoma, Radiotherapy

## Abstract

Male breast carcinoma (MBC) is rarely encountered in clinical practice. Due to its paucity, our knowledge of MBC only rely on small or single-institutional studies and sporadic cases. The current guidelines for MBC are extrapolated from its female counterparts Rudlowski (Breast Care (Basel) 3(3):183–189, 2008). Nowadays, MBC is actively studied and viewed as a potentially different entity on the aspects of etiology, biological behavior and prognosis. Thus, special treatment strategy guidelines should be established for MBC. Additionally, advance in the systemic chemotherapy and hormonal therapy also contribute to the local control. The indication of radiotherapy need to be clarified and over-treatment should be avoided. Here we present two cases of MBC in which radiotherapy help to sustain a satisfactory disease free survival. Our cases will provide valuable experience for identifying the role of radiotherapy in MBC.

## Background

MBC is exceedingly rare, composing less than 1 % of all the breast cancer [1]. Despite increasing incidence in recent years [2], many aspects such as best intervention, prognostic factors and so on are still unclear. Here, we present two rare cases of MBC. In the former, adjuvant radiotherapy contributed to prevention of local relapse. In the latter, radiotherapy was effective in controlling the recurrence of chest wall and achieved a good disease free survival (DFS) of at least 28 months.

## Case presentation

### Case 1

A 53-year-old man presented in January, 2011 with a history of painless lump of left breast for about 5 months. On physical examination, a hard lump measuring about 3.0 × 2.5 cm was palpated in the left breast and an enlarged, irregular lymph node of 2.0 × 1.5 cm was palpated in the left axilla. The breast ultrasound showed a 3.33 × 2.07 cm hypoechoic lesion in the left breast with blood flows and a 1.61 × 1.2 cm lymph node without normal structure. BI-RADS: 4b. The following Mammotome needle biopsy of left breast lump revealed invasive ductal carcinoma (IDC) with immunohistochemical results of ER (40 %+), PR(−), Her-2(−). Relevant staging examination ruled out distant metastasis. Thus, a male breast IDC of IIB, cT2N1M0, was diagnosed.

First, the patient received neoadjuvant chemotherapy of five cycles of TAC (Docetaxel, Adriamycin and Cyclophosphamide) and attained a near complete remission response. On 4th May, 2011, modified radical mastectomy (MCM) was performed and definitive pathology showed only a little residual ductal carcinoma in situ with partial microinvasion. Left axillary dissection proved negative for nodal metastases (0/29). The tumor bed was 1.5 × 1.1 × 1.0 cm and immunohistochemistry showed ER (40 %+), PR(−), Her-2(−). Then he received a cycle of TAC followed by adjuvant radiotherapy to left chest, supra and subclavicular region with a dose of 50 Gy/25f. Endocrine therapy was declined by the patient. To date, he lives a life of good quality and a 3-year DFS has been attained.

### Case 2

A 60-year-old man presented in April, 2010 with 1-month history of lump in right breast. Breast ultrasound demonstrated a 1.67 × 1.19 cm hypoechoic mass in the right breast. The subsequent biopsy demonstrated breast carcinoma. Then he received MCM in another hospital. The postoperative pathology showed IDC of grade II, with component of small tube carcinoma and right axillary lymph node metastases (2/14). Immunohistochemistry showed ER(−), PR(−), Her-2(−). He was staged as IIA, pT1N1M0. Then he received 6 cycles of chemotherapy but didn’t receive radiotherapy. 15 months later, recurrence of chest wall occurred and local resection was performed. The pathological results were as followings: IDC with invasion of pectoral muscle, vascular and nerve tissue, Ki-67 (+30 %), ER (+60 %), PR(−), Her-2(−). The borders were positive. The patient took tamoxifen but refused radiotherapy. 4 months later, a 2.0 cm × 2.0 cm mass appeared on the chest wall near the previous recurrent site. Then radiotherapy with a dose of 70 Gy/35f to the recurrence and 50 Gy/25f to the chest wall was carried out and the recurrent lesion disappeared. To date, DFS has reached to 28 months.

## Discussion

MBC is rather rare, with a predilection of elder men [3]. Despite great improvement in breast cancer treatment recently, MBC benefit less than FBC [4]. According to Surveillance, Epidemiology and End Result (SEER) data analysis, survival of breast cancer patients between 1996 and 2005 showed a 42 % decrease of breast cancer-specific death in female, but only a 28 % decrease in male [4, 5]. Comparing with female counterpart, MBC demonstrate more advanced stage-related tumor features such as 40 % stage III or IV disease [6] and 40–55 % axillary lymph node metastasis at first visit [7] and more tumor size >2.0 cm [8]. Interestingly, MBC manifested less advanced biology-related variables such as high tumor grade and ER or PR negativity [8]. IDC is the most common pathological type of MBC [9]. According to the multivariate analysis by Yoney et al. [9], lymph node metastasis was significantly associated with a poor DFS and overall survival (OS).

Recently, MBC is increasingly recognized as a different disease from FBC. Research have been initiated to clarify specific biomarkers of MBC pathogenesis. Genome-wide microarray analysis revealed miR-10a, miR-10b, miR-125b, miR-126 and miR-191 were underexpressed in MBC samples while miR-26b, miR-135b and miR-607 were overexpressed comparing with gynecomastia samples. As a target protein of miR-126, VEGF overexpressed correspondingly, partly contributing to the angiogenesis and progression of MBC. Comparing with FBC, 4 miRNAs overexpressed and 13 underexpressed in MBC [10]. Compared to gynecomastia, prolactin receptor expression was remarkably higher in MBC [4, 11]. Survivin and COX-2 were expressed in a substantial proportion of MBC individuals [4, 12]. In the near future, more specific miRNAs and genes will be identified in involvement of MBC development and as novel therapeutic target consequently.

Due to the lack of adequate breast tissue, MBC tends to invade the pectoralis major muscle. MCM is recommended as the standard surgical protocol to achieve R0 resection [13]. Sentinel lymph node (SLN) analysis seemed to be reliable in MBC [8]. In early-staged patients, breast conserving surgery might be feasible [4, 14].

In the past, radiotherapy was performed for all the postoperative MBC patients in view of inadequate surgical margin due to the lack of breast tissue [13, 15]. Yu et al. also indicated that postmastectomy radiotherapy (PMRT) bring significantly better local relapse free survival rather than OS benefit, especially for individuals with high risk factors such as ≤2 mm or unknown surgical margin, advanced stage and lymph node metastasis [16]. Now, PMRT indication in MBC follow recommendations for FBC, especially for those with axillary nodal involvement [17]. Besides, more MBC were inclined to PMRT than FBC for higher possibility of skin and nipple involvement [18, 19]. Korde et al. considered that retroareolar tumor or muscle invasion were also PMRT indication in MBC [8]. These two cases should be subjected to PMRT due to the involvement of axillary lymph nodes. The timely radiotherapy after MCM contributed to long-term DFS in the first patient. However, in the second case who didn’t receive PMRT, chest wall recurrence took place 15 months after surgery. The first 2 years was the peak of recurrence [9]. Radiotherapy after recurrence also played a critical role in local control. According to a 20-year survival data for PMRT in MBC, stage III disease could obtain OS benefit from PMRT whereas stages I and II could not. However, the results of this retrospective research was questioned for adverse long-term effects in earlier stages caused by obsolete irradiation techniques impaired the OS benefits [20]. Advanced radiotherapy techniques nowadays will maximize the dose to target volume and minimize the dose of normal organs, which might transform to the OS benefit for early-staged MBC patients.

90 % MBC cases express hormonal receptor. Thus, hormonal treatment is a crucial part of the management strategy [21]. Tamoxifen contributes to improved DFS and OS [22]. The application of aromatase inhibitors in MBC is still worthy of scrutiny [2]. The main reason is that testicular production of estrogen is independent of aromatase, composing about 20 % circulating estrogen [2, 23]. Furthermore, aromatase inhibitors has been reported to induce the increase of testosterone level providing more substrate for the production of estrogen [2, 24]. However, there is a high rate of discontinuation of tamoxifen in MBC due to one or more toxicity. Most common toxic effects are sexual dysfunction and weight gain [25]. In the second case, Tamoxifen alone failed to prevent the chest wall relapse while radiotherapy exerted favorable effect in local control. Because most of MBC patients are ER positive and older, adjuvant chemotherapy can benefit MBC with high risks such as young age, endocrine-nonresponse, high tumor grade and multiple axillary lymph node involvement [8].

Because of its rarity, only limited reports of small samples or single institute can be reviewed [26]. Cutuli et al. analyzed 489 MBC cases in their institute and revealed the similar outcomes to FBC after early diagnosis and wide application of adjuvant treatments such as radiotherapy, hormonal and chemotherapy [27]. However, biological differences between MBC and FBC should be emphasized and the treatment guidelines should not simply extrapolate FBC algorithms. In the future, collaboration of multiple institutions should be initiated and longer follow-up are fundamental for the full-scale research of this entity [26].

## Conclusion

MBC is now recognized as an entity with a potentially different biology and special treatment algorithms for MBC should be established in the near future. Radiotherapy indication for MBC patients need to be identified. Collaborative clinical trials are critical to clarify the optimal treatment for MBC.

## Consent

Written informed consent was obtained from the patient for publication of this case report and accompanying images. A copy of the written consent is available for review by the Editor-in-Chief of this journal.

## Abbreviations

DFS: disease free survival
ER: estrogen receptor
FBC: female breast carcinoma
IDC: invasive ductal carcinoma
MBC: male breast carcinoma
MCM: modified radical mastectomy
OS: overall survival
PMRT: postmastectomy radiotherapy
PR: progesterone receptor
SEER: surveillance, epidemiology and end result
SLN: sentinel lymph node
